# Quantification of Diabetes Comorbidity Risks across Life Using Nation-Wide Big Claims Data

**DOI:** 10.1371/journal.pcbi.1004125

**Published:** 2015-04-09

**Authors:** Peter Klimek, Alexandra Kautzky-Willer, Anna Chmiel, Irmgard Schiller-Frühwirth, Stefan Thurner

**Affiliations:** 1 Section for Science of Complex Systems, Medical University of Vienna, Vienna, Austria; 2 Gender Medicine Unit, Medical University of Vienna, Vienna, Austria; 3 Main Association of Austrian Social Security Institutions, Vienna, Austria; 4 Santa Fe Institute, Santa Fe, New Mexico, United States of America; 5 IIASA, Laxenburg, Austria; University of California San Diego, UNITED STATES

## Abstract

Despite substantial progress in the study of diabetes, important questions remain about its comorbidities and clinical heterogeneity. To explore these issues, we develop a framework allowing for the first time to quantify nation-wide risks and their age- and sex-dependence for each diabetic comorbidity, and whether the association may be consequential or causal, in a sample of almost two million patients. This study is equivalent to nearly 40,000 single clinical measurements. We confirm the highly controversial relation of increased risk for Parkinson’s disease in diabetics, using a 10 times larger cohort than previous studies on this relation. Detection of type 1 diabetes leads detection of depressions, whereas there is a strong comorbidity relation between type 2 diabetes and schizophrenia, suggesting similar pathogenic or medication-related mechanisms. We find significant sex differences in the progression of, for instance, sleep disorders and congestive heart failure in diabetic patients. Hypertension is a highly sex-sensitive comorbidity with females being at lower risk during fertile age, but at higher risk otherwise. These results may be useful to improve screening practices in the general population. Clinical management of diabetes must address age- and sex-dependence of multiple comorbid conditions.

## Author Summary

We quantify for the first time age- and gender-dependent relative risks for each possible comorbidity of type 1 and 2 diabetes in a nation-wide claims dataset containing almost two million patients, and test whether the association may be consequential or causal. This study therefore contains almost 40,000 single clinical measurements, all with the maximum patient number available in an entire country. We confirm the relation between diabetes and Parkinson's disease, and find different progression routes of mental disorders in type 1 and type 2 diabetics. Among many other results, we also report significant gender differences in the progression of congestive heart failure, sleep disorders, hypertension, and hyperlipidemia. This work provides the first complete statistical description of all diabetic comorbidities and their dependence on patient age and sex. These results may be of immediate use to improve screening practices and therapy of diabetic patients due to more accurate diagnosis and treatment of important comorbidities.

## Introduction

Diabetes is a global pandemic disease. The world-wide number of adult diabetes patients doubled over the last three decades to approximately 350 million as of 2010, and is expected to double again until 2030 as a result of population ageing and a shift to western lifestyle patterns in developing countries [1]. Diabetes comprises a heterogeneous group of disorders with the most prominent types being type 1 (DM1) and 2 diabetes (DM2). These disorders have different pathophysiology and phenotype; the exact underlying mechanisms, their interplay finally leading to manifestation, progressions of the diseases, and their complications are still unclear. Diabetes is related to a large number of comorbid diseases, including but not limited to vascular complications [2], renal failures [2], neuropathy [2], heart diseases [3, 4], cognitive disorders [5, 6], retinopathy [7], and hypertension [8]. Each of these comorbidities opens up a unique direction of research. Following the methodological approach developed in this work, thousands of such relations can be investigated in parallel. Besides studying the individual diabetic comorbidities and how they depend on patient age and gender, this allows to compare the strength of these relations among each other and to rank them according to their significance.

Nation-wide collections of physician and hospital claims data allow to explore the health state of an entire country’s population with unprecedented precision and scale [9]. To exploit the full potential of ‘big data’ for medical sciences the development of novel, quantitative methods to extract clinically relevant features from large datasets of electronic health records (EHR) is necessary. First efforts in this direction have proven to be extremely fruitful by developing or improving data-driven comorbidity indices to predict mortality rates [10], or by studying healthcare utilization and outcome measures of specific patient cohorts [11]. Large-scale analyses of comorbidities using EHR data have demonstrated that human disease phenotypes can be related to each other in highly connected networks with strong pairwise correlations between diseases [12, 13, 14, 15]. In this work we develop a new quantitative framework to measure age- and gender-dependent relative risks for all possible comorbidity relations for DM1 and DM2 using medical claims data from almost two million people. We introduce tests to assess the significance of the comorbidity relations, the influence of sex, and whether diabetes is more likely to be a diagnosed before or after the other disease.

## Materials and Methods

### Data

A research database of the Main Association of Austrian Social Security Institutions containing pseudonymised claims data of *all* persons receiving care in Austria between January 1st, 2006 and December 31st, 2007 is used [16]. The data gives a comprehensive, nation-wide picture of the medical condition of most of the approximately 8.3 million Austrians. The patient collective was formed by extracting all persons receiving inpatient care in 2006 or 2007. We identified patients being diagnosed with DM1 or DM2 (ICD10 codes E10 and E11). Patients who died in 2006 or 2007 were removed. In this way 16 667 DM1 patients (8 355 males and 8 312 females) and 105 904 with DM2 (50 596 males and 55 308 females) were selected. The total sample of inpatients used in this study consists of 1 862 258 patients (1 064 952 females and 797 306 males). From these patients we know their year of birth, sex, ATC codes of all their prescriptions, and the ICD codes of all their diagnoses (main- and side-diagnoses).

**Co-occurrence analysis/ relative risks for comorbidities**. For the occurrences of each diagnosis *x* (ICD10, three-digit-level) a patient-age-resolved cross tabulation with the occurrences of DM1 and DM2 is performed. Symptoms, injuries, pregnancies, and external causes and factors of morbidity were excluded. We therefore test 1 051 diagnosis (ICD10 codes ranging from A01 to N99) for their co-occurrence with diabetes. The patients are grouped by their age in five-year intervals and by their gender. Patients older than 95 have been excluded. We test 1 051 possible comorbidities for 19 age groups for DM1 and DM2, giving 39 938 tests. For each diagnosis and age interval a contingency table is built. If each entry in the table is greater than 10, *relative risks RR*_1(2)_(*x*,*t*) are computed, a chi-squared test is performed and *p*-values are calculated for rejecting the null hypothesis that co-occurrence of the diagnosis with DM1 or DM2 is independent. This leads to a multiple hypothesis testing problem for each age group where 1 051 hypotheses are tested in parallel. To correct for these multiple comparisons we apply the Benjamini-Hochberg procedure [17] to control for the false discovery rate *α*. This procedure is a multiple comparison correction where the value of *α* gives the expected probability that a null hypothesis is incorrectly rejected. For example, if 100 comorbidities are identified with a false discovery rate *α* of *α* = 0.01, the expected number of false positives among these comorbidities is one. If there are less than ten co-occurrences or the results are not significant, the relative risk is set to one. For the co-occurrence analysis we use both the main and the side diagnoses of each patient.

**Validation of the co-occurrence analysis.** To validate the results of the co-occurrence analysis we compile a list of major known diabetic complications from different literature sources [18, 19, 20]. These lists are based on hand curated collections of diabetic comorbidities, some of them validated using EHR data [19, 20]. These studies disagree on the exact list of ICD codes for diabetic complications, but each list focusses on cardiovascular, renal, and ophthalmic comorbidities. The ICD codes that are listed as diabetic complications in *each* of these studies are therefore used to validate our co-occurrence analysis, see Table 1. Note that, for example, mental disorders like depression or pancreatic cancer, both well-known diabetic comorbidities [5, 6, 21], are not included in any of these studies. Nevertheless, a valid method to detect comorbidities is supposed to pick up a substantial number of the diagnoses listed in Table 1, among other comorbidities. We will therefore be interested in the recall *R*(*α*) as a function of the false discovery rate *α*. *R*(*α*) is the probability that a diabetic comorbidity listed in Table 1 is also identified by our co-occurrence analysis at a given level of *α*.

**Table 1:** A list of major well-known diabetic comorbidities that is used to validate the results of the co-occurrence analysis.

ICD10	Diagnosis
G45	Transient cerebral ischemic attacks and related syndromes
I10	Essential (primary) hypertension
I20	Angina pectoris
I21	ST elevation (STEMI) and non-ST elevation (NSTEMI) myocardial infarction
I24	Other acute ischemic heart diseases
I47	Paroxysmal tachycardia
I50	Heart failure
I60	Nontraumatic subarachnoid hemorrhage
I61	Nontraumatic intracerebral hemorrhage
I62	Other and unspecified nontraumatic intracranial hemorrhage
I65	Occlusion and stenosis of precerebral arteries, not resulting in cerebral infarction
I66	Occlusion and stenosis of cerebral arteries, not resulting in cerebral infarction
I67	Other cerebrovascular diseases
I70	Atherosclerosis
I71	Aortic aneurysm and dissection
I72	Other aneurysm
I73	Other peripheral vascular diseases
I74	Arterial embolism and thrombosis
M86	Osteomyelitis
N17	Acute kidney failure
N18	Chronic kidney disease
N19	Unspecified kidney failure

**Sex ratio**. The *sex ratio SR*(*x*,*t*) is related to the quotient of the percentage of female and male diabetes patients in age group *t* that also have diagnoses *x* or are prescribed a medication *x*. Denote the number of male (female) DM1 and DM2 patients in age group *t* by *D*_*m(f)*_(*t*) and the number of male (female) diabetes patients who also have diagnoses or medication *x* by *D*_*m(f)*_(*x*,*t*). The *sex ratio SR*(*x*,*t*) is then related to the logarithmic quotient of the percentage of female and male diabetes patients who also have diagnoses *x*,

$$SR(x,t)=log[\frac{1+\frac{D_{f}(t)}{D_{f}(x,t)}}{1+\frac{D_{m}(t)}{D_{m}(x,t)}}].$$ (1)

A value of *SR*(*x*,*t*) that is close to zero indicates that the co-occurrence of the diagnosis or medication *x* with diabetes is equally likely for males and females. Positive (negative) values of *SR*(*x*,*t*) indicate that the co-occurrence is more likely for females (males). To assert the statistical significance of nonzero *SR*(*x*,*t*) values we build a contingency table for all diabetes patients of a given age group *t*. The table contains the two variables sex and co-occurrence with diagnosis/medication *x*. If the null hypothesis of statistical independence of these two variables cannot be rejected in a chi-squared test using a *p*-value of *p* = 0.05 the sex ratio is set to zero, *SR*(*x*,*t*) = 0.

**Lead/lag indicator.** The lead/lag indicators assess whether patients with diagnoses *d*_*i*_ are more likely to be later diagnosed with another disease *x*, the lead indicator *I*_*lead*_(*d*_*i*_,*x*), or whether it is more likely that people having diagnoses *x* will be diagnosed with diabetes, the lag indicator *I*_*lag*_(*d*_*i*_,*x*). There exist several known biases in EHR data that need to be addressed in the definition of these indicators [22]. (i) The first occurrence of a coding of a diagnosis in the EHR data will typically not correspond to the true initial diagnosis of the disease. (ii) The data only spans two years, which may not be enough to observe the manifestation of diabetic complications directly.

We use the following methodology to measure the lead/lag indicators and adjust for these known biases. Let us consider the lead indicator *I*_*lead*_(*d*_*i*_,*x*) that measures if the diagnosis *x* is typically made after the diabetes diagnosis. Given the limitations of our data, we cannot observe the typical time between the manifestations of the two diseases. We can, however, measure whether there is a tendency that *x* will be diagnosed in a patient that already had a prior diabetes diagnosis. As opposed to the co-occurrence analysis, it is crucial for the lead/lag analysis to distinguish between main- and side diagnoses. To this end we consider the probability that a male (female) patient has a diabetes diagnosis (main or side diagnosis) in year *t*_1_, and a main-diagnosis *x* in year *t*_2_, but no diagnosis of *x* in *t*_1_ (main or side diagnosis). Denote this probability by *p*_*m*(*f*)_(*x*,*t*_2_|*d*_*i*_, ¬*x*,*t*_1_) for males (females). This number over-estimates the true effect size, since some cases where a patient does not have diagnosis *x* in year *t*_1_ might be due to inaccuracies in the coding or incompleteness of the data, in particular with respect to unknown pre-existing conditions. However, we assume that these errors are not systematic in the sense that they are equally likely to influence the data for year *t*_1_ and *t*_2_. If there is no true temporal ordering in the onsets of *d*_*i*_ and *x*, the value of *p*_*m*(*f*)_(*x*,*t*_2_|*d*_*i*_, ¬*x*,*t*_1_) just measures noise due to incomplete or inaccurate data. But this is equally true for the probability that diagnosis *x* does not occur for a patient in year *t*_2_, given that she(he) has both diagnosis *d*_*i*_ and *x* in *t*_1_, the probability *p*_*m*(*f*)_(*x*,*t*_1_|*d*_*i*_, ¬*x*,*t*_2_). If there is a substantial tendency that *x* is diagnosed *after* the onset of *d*_*i*_, however, these two probabilities are likely to differ. The lead indicator *I*_*lead*_(*d*_*i*_,*x*) is therefore given by

$$I_{lead}(d_{i},x)=p(x,t_{2}|d_{i},\neg x,t_{1})-p(x,t_{1}|d_{i},\neg x,t_{2}).$$ (2)

The lag indicator *I*_*lag*_(*d*_*i*_,*x*) is constructed in analogy to the lead indicator *I*_*lead*_(*d*_*i*_,*x*) and by exchanging the roles of *d*_*i*_ and *x*,

$$I_{lag}(d_{i},x)=p(d_{i},t_{2}|\neg d_{i},x,t_{1})-p(d_{i},t_{1}|\neg d_{i},x,t_{2}).$$ (3)

If the frequency of the diagnosis *x* itself is very small already a very small number of events might lead to comparably large indicator values for *I*_*lead*_(*d*_*i*_,*x*) and *I*_*lag*_(*d*_*i*_,*x*). We therefore exclude diagnoses *x* from the analysis if they have less than a threshold of *z* male or female patients that also have *d*_*i*_ in *t*_2_. In the following we set *t*_1_ = 2006 and *t*_2_ = 2007. For the lag indicator for DM1 we exclude all patients older than 30.

Finally, a statistical test is developed to assess the significance of positive values for *I*_*lead*_(*d*_*i*_,*x*) and *I*_*lag*_(*d*_*i*_,*x*). Surrogate data is created by keeping the list of diagnoses for each patient fixed and by shuffling the information about the year when the diagnoses were made. Assume that patient *p* has *n*_*p*_ diagnosis {*x*_*i*_} made in the years {*τ*_*i*_} with *i* ∈ {1,…, *n*_*p*_}. The surrogate data is constructed by replacing {*τ*_*i*_} by a random permutation of itself. This procedure is repeated 1 000 times and the lead and lag indicators are computed for each surrogate dataset. We test the null hypothesis that the values for the lead and lag indicators observed in the data are as large as one would expect for indicator values taken from the surrogate data, where the temporal information has been randomly shuffled. The *p*-value for each lead and lag indicator is the probability of obtaining the observed values for *I*_*lead*_(*d*_*i*_,*x*) and *I*_*lag*_(*d*_*i*_,*x*) from the surrogate data. The null hypothesis is rejected if *p*<0.01, that is if out of 1 000 surrogate datasets less than ten give indicator values that are larger than the observed values.

A significant value of the lead indicator *I*_*lead*_(*d*_*i*_,*x*) suggests that the incidence of disease *x* is more likely in patients with pre-existing diabetes compared to the incidence of diabetes in patients with pre-existing disease *x*. A significant value of the lag indicator *I*_*lag*_(*d*_*i*_,*x*), on the other hand, suggests that diabetes is typically incident in patients already diagnosed with *x*. A similar approach to study lead/lag behavior between diseases, but without a test for statistical significance of the results, was proposed for networks of comorbid diseases [12].

## Results/Discussion

**Fig 1:**
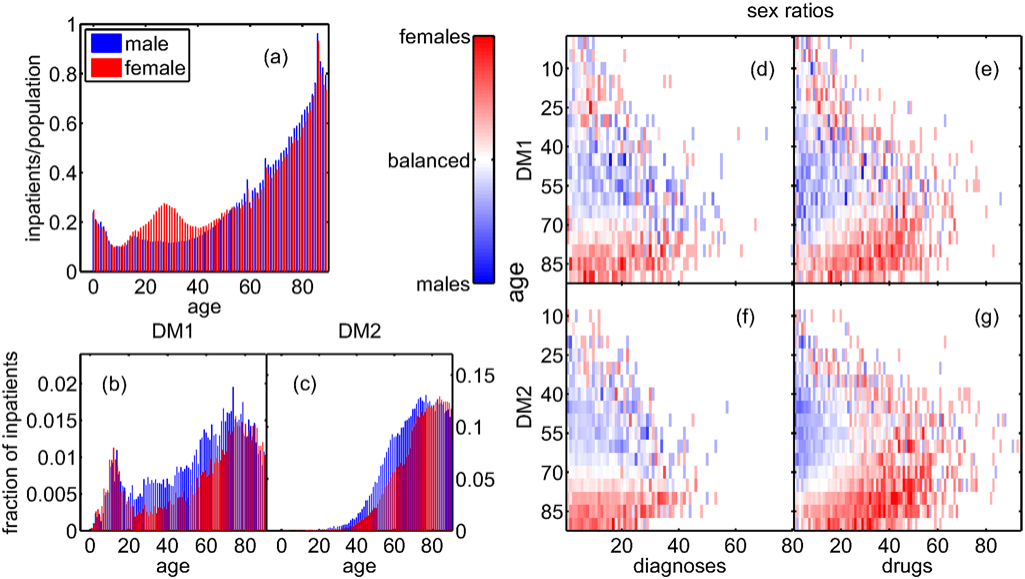
The fraction of inpatients in the entire population as a function of age is similar in males and females, except for an excess of females at the age around 30, most likely related to giving birth (a). After a peak in early childhood, the fraction of inpatients increases to levels of above 80% in older age. The bulk of male (female) (b) DM1 and (c) DM2 patients is aged around 60 (70), for DM1 patients there is a second peak around age 10. The sex ratio *SR(y*,*t)* is shown for DM1 (d,e) and DM2 (f,g) patients and the number of their diagnoses (d,f) and their prescriptions (e,g). For patients younger than 60, with a comparably high number of comorbidities, female patients have less diagnoses but take more drugs than males.

Fig. 1(a) shows the fraction of male and female inpatients of the entire population as a function of age. The inpatient fractions are around 20% for children under five, then drop to 10–15% for ages around ten, and from then on rise to more than 80% for 80 year-old patients, with an additional peak for females of age around 30, most likely due to child birth. With increasing patient age the inpatient sample becomes increasingly representative of the entire population. Fig. 1(b) shows the fraction of male and female DM1 inpatients as a function of age. The distributions have a first peak around the typical onset-age of ten for both male and females, and a second peak for ages 60 (70) for males (females). Fig. 1(c) shows the fractions of inpatients diagnosed with DM2 as a function of age, with comparably few patients below age thirty, and the bulk of male (female) patients concentrated around age 60 (70).

Fig. 1 shows the sex ratio *SR*(*x*,*t*) for DM1 patients and their number of diagnoses (d) and received drugs (e); (f) and (g) show the same for DM2 patients. Up to an age of 60 there is an excess of male patients, for older patients there is an excess of females. For drugs there is a male excess only for age up to 60 and for less than 10–20 drugs. For older age and a larger number of drugs there is an excess of female patients. Females below age 60 have fewer diagnoses than males, but especially those with a large number of diagnoses have more prescriptions than males. After age 60, females outweigh males in both diagnoses and prescriptions.

**Fig 2:**
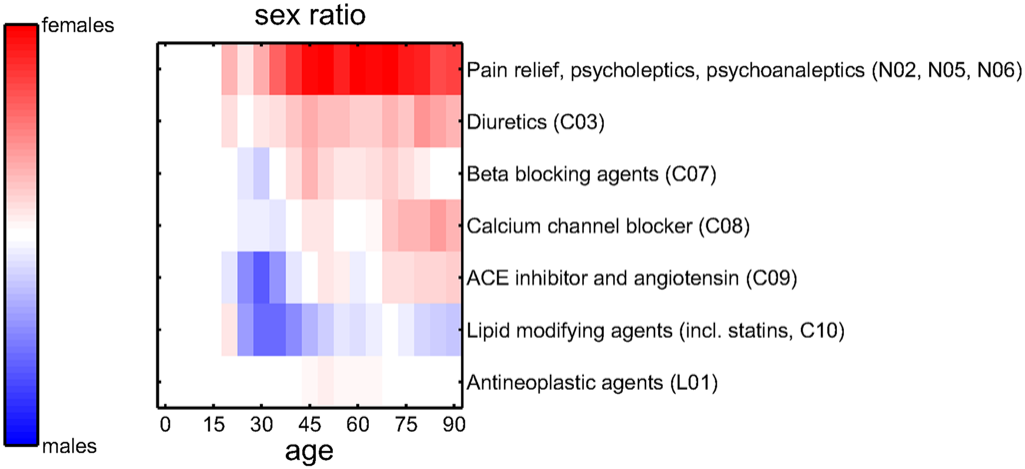
Sex ratios for the numbers of prescriptions of selected therapies on 3-digit-level ATC codes. Results for medications related to pain relief, psycholeptics, and psychoanaleptics (N02, N05, N06) and diuretics (C03) are dominated by females at all ages. Beta blocking agents (C07), calcium channel blocker (C08), and ACE inhibitors (C09) show an excess of males at ages around 30, but a female excess at older ages. Lipid modifying agents (C10) show an excess of males, the sex ratio for antineoplastic agents (L01) is almost balanced.

The sex ratios for selected groups of medications are shown in Fig. 2. Drugs are classified according to their 3-digit-level ATC codes. The sex ratios for drugs for pain relief, psycholeptics, and psychoanaleptics (N02, N05, N06), but also for diuretics (C03) are dominated by females at all ages. Beta blocking agents (C07), calcium channel blocker (C08), and ACE inhibitors (C09) show an excess of males at ages around 30, but a female excess at older ages. Lipid modifying agents (C10) show an excess of males, whereas the gender ratios for antineoplastic agents (L01) are almost balanced.

Each diagnosis where the null hypothesis of statistical independence with either DM1 or DM2 can be rejected with a given value of the false discovery rate in at least one of the age groups is identified as a comorbidity. The results of the co-occurrence analysis are validated by considering the recall *R*(*α*) for the major diabetic comorbidities from Table 1. A false discovery rate of *α* = 0.001 gives a list of 75 significant comorbidities and a recall of *R*(*α* = 0.001) = 0.59. For *α* = 0.01 we retrieve 123 comorbidities with recall *R*(*α* = 0.01) = 0.73, and for *α* = 0.1 we get 297 comorbidities with recall *R*(*α* = 0.1) = 1. In the following we choose a threshold of *α* = 0.01. The expected number of false positives among these comorbidities is 1.23. Note that for this threshold we pick up several diseases that are very closely related to those major diabetic complications that we do not retrieve. For example, we do not pick up the subarachnoid, intracerebral, and intracranial hemorrhages (I60-I62), but we retrieve cerebral infarctions (I63) and other strokes (I64). Similarly at this threshold we do not retrieve aneurysms (I71–72), but artherosclerosis and other peripheral vascular diseases (I70, I73). We identify occlusion and stenosis of cerebral arteries (I66) instead of precerebral arteries (I65).

**Fig 3:**
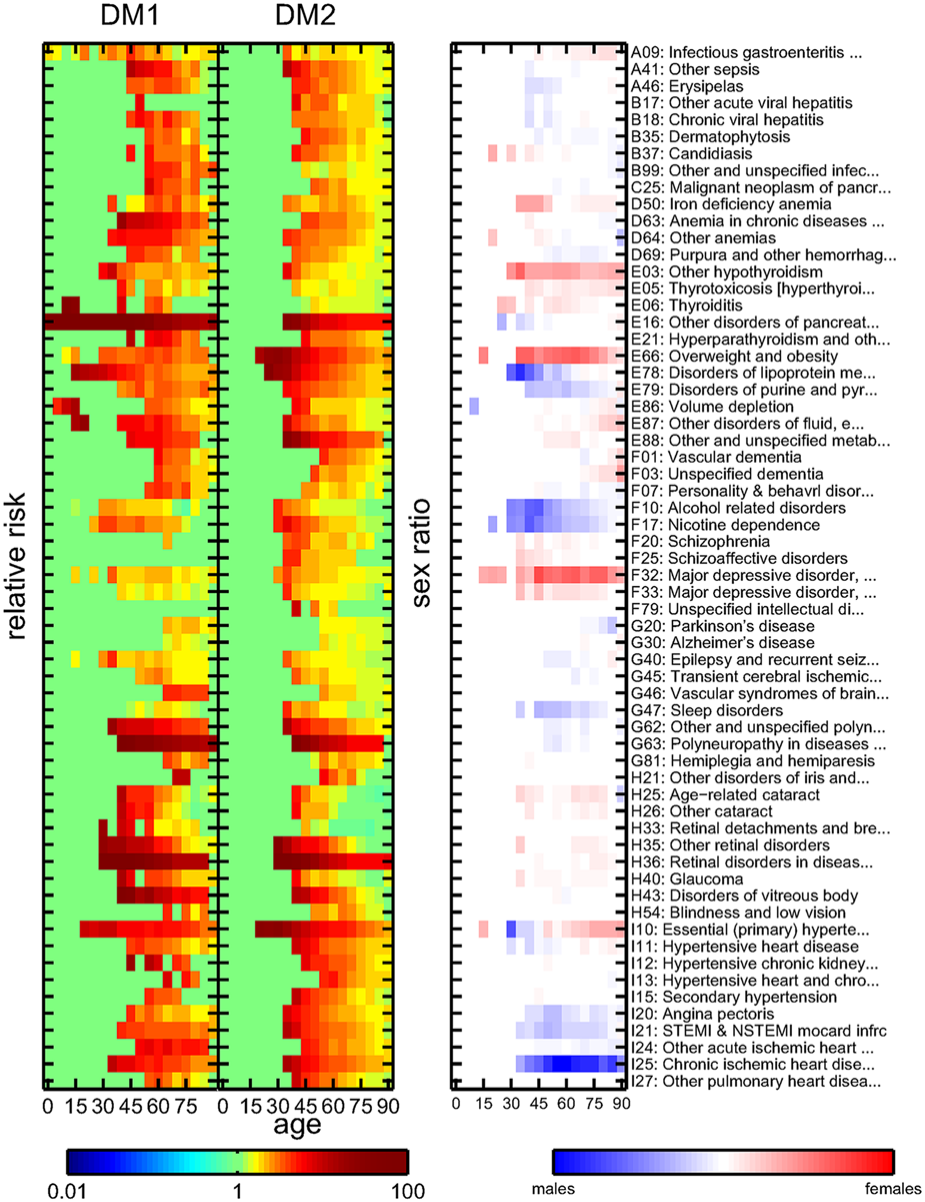
Relative risks for DM1 (left column) and DM2 (middle column) patients, and sex ratios (right column) for core comorbidities using a false discovery rate of *α*<0.01 and an ICD code from the range A01-I27. Color encodes the values of the risks and sex ratios.

**Fig 4:**
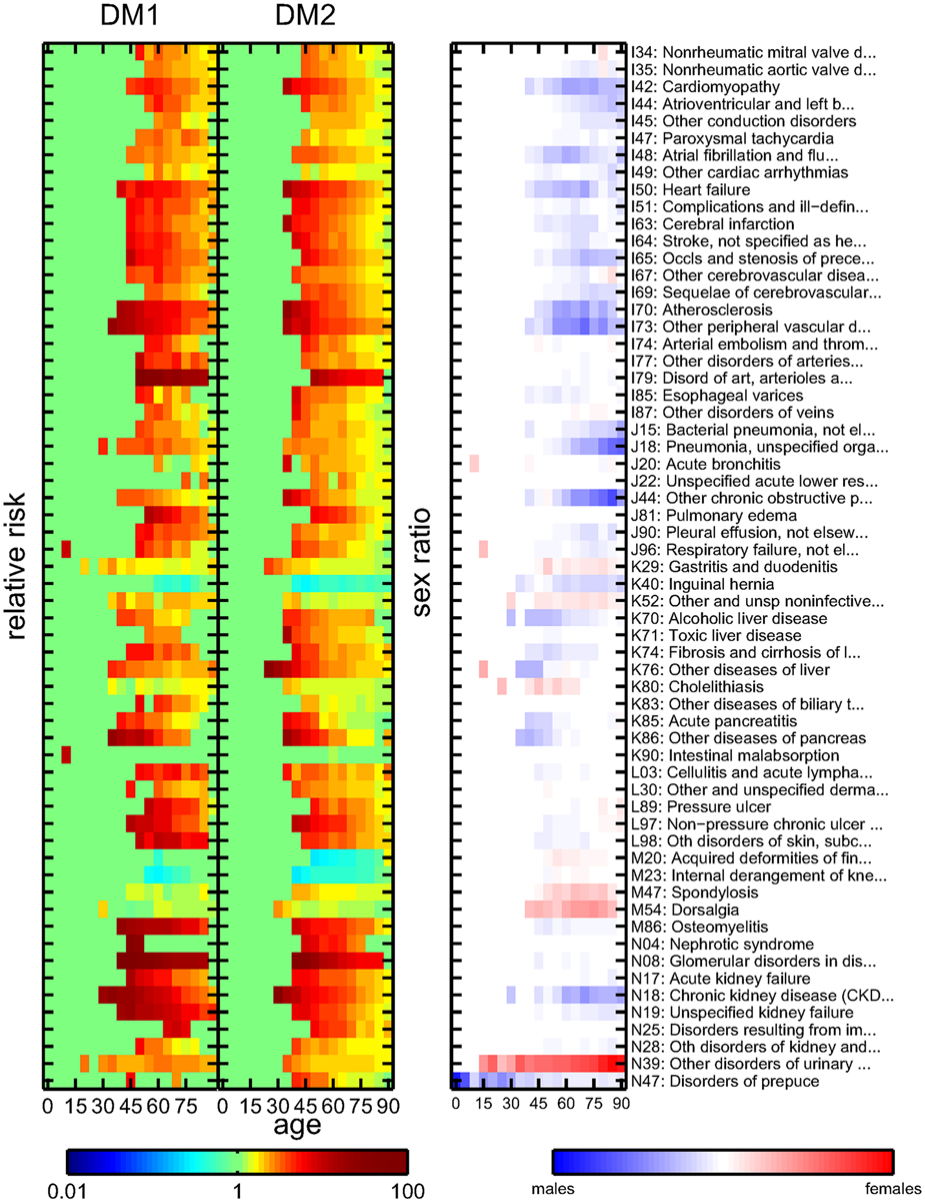
Relative risks for DM1 (left column) and DM2 (middle column) patients, and sex ratios (right column) for core comorbidities using a false discovery rate of *α*<0.01 and an ICD code from the range I34-N99. Color encodes the values of the risks and sex ratios.

The results are summarized in Fig. 3 and Fig. 4, with the left columns showing the DM1 relative risk *RR*_1_(*x*,*t*), the middle columns the DM2 relative risk *RR*_2_(*x*,*t*), and the right columns the sex ratio *SR*(*x*,*t*). The comorbidities are also listed in the supplement, S1 Table, along with relative risks, *p*-values, and patient ages for the age group with the smallest *p*-values for DM1 and DM2, respectively. In the following we refer to these values whenever referring to the relative risks of a diagnosis with a 95% confidence interval (CI).

Lead/lag behavior is identified for male and female DM1 and DM2 patients if the null hypothesis that the observed indicator values for *I*_*lead*_(*d*_*i*_,*x*) and *I*_*lag*_(*d*_*i*_,*x*) can be obtained from randomized surrogate data can be rejected with a *p*-value of *p*<0.01. The threshold *z* is set to *z* = 50 for DM1 and DM2. Table 2 shows diagnoses which have been identified as either leading or lagging for male or female DM1 or DM2 patients.

**Table 2:** Diagnoses are shown which have been identified in the lead/lag analysis.

ICD	diabetes type	sex
*Diabetes leads (comes before other disease)*		
A41 Other sepsis	2	F
C25 Malignant neoplasm of pancreas	2	F
C34 Malignant neoplasm of bronchus and lung	2	M
E16 Other disorders of pancreatic internal secretion	2	M
F03 Unspecified dementia	2	F
F32 Depressive episode	1	M
G45 Transient cerebral ischemic attacks and related syndromes	1	F
I25 Chronic ischemic heart disease	1	F
I46 Cardiac arrest	2	M+F
I48 Atrial fibrillation and flutter	1	M
I50 Heart failure	1	F
I50 Heart failure	2	M
J18 Pneumonia, unspecified organism	2	M
L89 Decubitus ulcer	2	M
M48 Other spondylopathies	2	M
N18 Chronic renal failure	2	F
*Diabetes lags (comes after other disease)*		
C44 Other and unspecified malignant neoplasm of skin	2	M
D40 Neoplasm of uncertain behavior of male genital organs	2	M
K81 Cholecystitis	2	M

For each diagnoses the order (if diabetes leads or lags), gender (‘F’ for females, ‘M’ for males) and diabetes type (1 or 2) where the relationship was detected are listed.

In the following we discuss results for individual comorbidities. Emphasis is put on comorbidities that have been disputed in the literature, or where the lead/lag analysis advances our understanding of them. Another important group of results consists of comorbidities for which we find a yet unknown degree of sensitivity to sex. In particular we find for several comorbidities a certain patient age where the sex ratio switches from an excess of one sex to an excess of the different sex for older ages; we will refer to these patient ages as ‘age switch’.

### Controversial comorbidity associations

**Parkinson’s disease.** In the literature there is no consensus on whether diabetes patients have a higher risk for Parkinson’s disease (PD), or if there is actually a lower risk or no relation at all. There are two large prospective studies finding an increased risk for PD in diabetes patients, one study finding no relation, and one study reporting lower risk of diabetes [23]. We find that PD is comorbid (2.3, CI 1.9–2.7 for DM1 and 1.5, CI 1.4–1.6 for DM2) with an excess of male patients. It has been suggested that surveillance bias may lead to the reporting of spurious positive correlations between PD and diabetes [23]. Given our patient cohort we can exclude this kind of bias. Note that the size of our patient cohort (1.8 million patients) is at least 10 times larger than the largest cohorts in previous studies on the relation between PD and diabetes [23, 24]. As potential mechanism of this association the involvement of insulin in the regulation of brain dopanergic activity has been proposed [25, 26]. Animal and in vitro studies have shown that insulin and dopamine may exert reciprocal regulation [26].

**Mental disorders.** Depression, schizophrenia, and schizo-affective disorders are also comorbid. While the relative risks for DM1 patients are highest in the age group 65–70 with values from 1.9–2.3 for these diseases, we find higher risks for DM2 patients at younger ages, e.g. a relative risk of 4.8, CI 3.3–7.0, for recurrent depressive disorders at age 35–40. We find that depression is usually incident in DM1 patients. From these results one may speculate that DM1 patients develop depressions because of the burden of the disease and the psychological distress of maintaining a good level of glycemic control. Depression in diabetic patients in general, DM1 and DM2, is dominated by females [5], so is the association between depression and overweight [27]. Indeed it is remarkable that depression and overweight as diabetic comorbidities show nearly the same age and sex dependence. A possible biological mechanism is that obesity increases the risk of increased insulin resistance, which may induce alterations in the brain which in turn increase the risk of depression [28]. Of importance are also psychological pathways, since the perception of being overweight increases psychological distress [29]. Diabetes has also been associated with the use of atypical neuroleptics in the treatment of schizophrenia [30]. The sex ratios for antipsychotics show a strong excess of female patients, see Fig. 2, which compares well with the female excess in the sex ratios for depression and schizophrenia. It is interesting to note that the comorbidity relations with schizophrenia and schizo-affective disorders stand out as much weaker for DM1 than for DM2 patients, when compared to all other results of the comorbidity analysis.

### Gender-specific results on comorbidities

**Endocrine and metabolic disorders.** While patients with thyroiditis, hypothyroidism, thyrotoxicosis, and obesity are predominantly female, disorders of the lipoprotein, purine, and pyrimidine metabolism tend to be found in males. Diabetic patients feature a two to three times higher increased risk of disorders of the thyroid gland, particularly those with autoimmune diabetes, a comorbidity relation that is strongly influenced by gender [31]. For volume depletion and disorders of fluid, electrolyte and acid-base balance there appears to be an age switch, from an excess of male patients for ages 20–40 to an excess of females in older age. Primary hypertension is a comorbidity with relative risks of 5.3 (CI 4.8–5.9) for DM1 and 9.5 (CI 8.8–10) for DM2. These switches may indicate an important impact of sexual hormones and of potential pregnancies but may also point to social factors related to sex-specific phases of life. The prescriptions of beta and calcium channel blocker, as well as ACE inhibitor show a sex-dependence very similar to hypertension, suggesting that these drugs are commonly used to treat hypertension, see Fig. 2. There is a strong excess of female patients in the prescriptions of diuretics, especially in elderly patients, whereas there is a strong excess of younger males being prescribed statins or other lipid modifying agents, the latter matching the sex ratio observed for hypercholesterolemia and hyperlipidemia. Note that our results make no statements about the combinations of antihypertensive drugs which are actually used in the treatment of individual patients.

**Infections and sepsis.** Bacterial and viral infections (gastroenteritis, erysipelas, pneumonia, osteomyelitis, hepatitis, dermatophytosis, candidiasis) show an excess of male patients with the exception of gastroenteritis and candidiasis, which are dominated by female patients. We find an excess of sepsis comorbidity which is strongest in male DM1 patients at the age around 50, with higher relative risks for DM1 (12, CI 8.2–18) than DM2 (2.7, CI 2.4–2.9).

**Epilepsy**. The increased risk for epilepsy (4.6, CI 3.1–6.9, for DM1 and 1.6, CI 1.4–1.7, for DM2) in young type 1 diabetics [32] may be linked to ketoacidosis as a two times higher risk of epilepsy was found in children and adolescents with metabolic acidosis [33]. A four times greater risk of DM1 was also described in young adults with epilepsy [34]. Both metabolic extremes, hypoglycemia and diabetic ketoacidosis, relate to EEG abnormalities in diabetic children which may increase risk of epilepsy.

**Congestive heart failure**. The Framingham heart study reported that diabetic women are more vulnerable to congestive heart failure (CHF, RR of 5.2, CI 4.7–5.9, for DM1, 3.8, CI 3.6–3.9, for DM2) than men [35]. However, subsequent cohort studies found no such sex differences [35, 36]. We find an excess of male patients and that diabetes is typically detected before CHF in females with DM1 and in males with DM2.

**Sleep disorders.** Sleep disorders are comorbid in DM1 (1.9, CI 1.5–2.4) and DM2 patients (2.3, CI 2.1–2.6). We find support for sex specific progression routes. It is known that DM2 and obstructive sleep apnea (OSA) present a vicious circle, with OSA exerting adverse effects on glucose metabolism and thereby increasing the risk for DM2 [37]. In patients with already existing DM2, on the other hand, there is a significant relationship between sleep-disordered breathing (SDB) and insulin resistance independent of obesity [38]. The fact that there is an excess of male patients in the comorbidity relation may be related to the higher prevalence of central adiposity and therefore OSA in men [37].

### Further comorbidities

**Pancreatic cancer.** There are higher relative risks for DM1 patients (8.6, CI 5.6–13) than DM2 patients (2.5, CI 2.1–2.8). The risks peak in the age range 50–70 with a balanced sex ratio. It has been shown that diabetic patients are at increased risk of pancreatic cancer with a pooled RR of approximately two compared to non-diabetics in a meta-analysis [21] with at least one year diabetes duration prior to diagnosis of pancreatic cancer [39]. Diabetes also leads the diagnosis of pancreatic and lung cancer.

**Behavioral and related disorders.** Nicotine dependence (3.3, CI 2.7–4.1, for DM1 and 2.8, CI 2.6–3.0, for DM2) and alcohol related disorders dependence (2.3, CI 1.7–3.2 and 2.1, CI 1.9–2.4) are comorbidities with relative risks peaking at ages 30–45, dominated by male patients. Alcoholic liver disease dependence (4.0, CI 2.7–5.7 and 2.6, CI 2.3–2.9) is also a male-dominated comorbidity. Toxic liver disease (2.8, CI 1.6–4.9 and 14, CI 8.5–23) and fibrosis and cirrhosis of liver (5.0, CI 3.7–6.6 and 2.4, CI 2.2–2.7) show also an excess of male patients. There tend to be higher risks for DM1 than DM2 patients, potentially outlining greater impact of chronic hyperglycemia than of overweight-related parameters of the metabolic syndrome. The relationship between alcohol consumption and DM2 has been shown to be dosage dependent. While moderate alcohol consumption is protective, dosages of more than 60g/day increase diabetes risk [40]. It is not possible to establish an alcohol-dosage dependent diabetes risk from our data.

**Cardiovascular diseases**. Identified comorbid diseases of the circulatory system include ischemic and pulmonary heart disease, cardiomyopathy, valvular disorders, tachycardia, as well as cerebrovascular diseases and diseases of the arteries and veins [2, 4, 41]. Comorbid diseases of the circulatory system show a consistent excess of male patients, including ischemic, pulmonary, and other heart diseases (cardiomyopathy, valvular disorders, tachycardia), as well as cerebrovascular diseases and diseases of the arteries and veins. The highest relative risks among cardiovascular diseases are found for acute ischemic heart diseases for DM1 patients (6.6, CI 5.2–8.3, compared to 3.1, CI 2.8–3.4, for DM2 patients) at ages higher than 60.

**Pulmonary diseases.** Pneumonia and acute bronchitis show increased relative risks for older ages (e.g. for pneumonia 2.7, CI 2.4–3.0, for DM1, 2.3, CI 2.1–2.4, for DM2). Chronic obstructive pulmonary disease (COPD) is led by diabetes (2.9, CI 2.5–3.5 and 2.2, CI 2.1–2.3). Diabetes is often identified as independent risk factor for lower respiratory tract infections [42]. Individuals with COPD are substantially more likely to have pre-existing DM [43], on the other hand lung function impairment in COPD is a risk factor for developing diabetes and insulin resistance [44]. Benign pleural effusion (3.4, CI 2.1–5.6 and 3.1, CI 2.5–3.9), representing a symptom of various underlying diseases, is dominated by males. In diabetic patients pleural effusion may be related to left ventricular dysfunction as described previously [45].

**Other comorbidities.** Iron-deficiency and anemia in chronic diseases show higher relative risks for DM1 (3.7, CI 3.0–4.6, and 6.3, CI 4.9–8.1) than DM2 (2.7, CI 2.4–2.9 and 2.8, CI 2.5–3.2) patients. Cataracts, retinal detachments, glaucoma, disorders of the vitreous body, and blindness are identified here with relative risks up to 200. The higher relative risks for DM1 compared to DM2 patients for retinopathies [7] at older age suggest a higher lifespan for type 1 diabetics. Chronic and acute kidney diseases, the nephrotic syndrome, and glomerular disorders are identified as comorbidities with an excess of male patients; relative risks range up to 128 for DM1 patients and 8.6 for DM2. There is an excess of female patients in the age range 20–40. Intestinal malabsorption (including celiac disease) shows elevated risks for ages 10–25 for DM1 (10, CI 6.3–17) with a weak female excess; there are no significant results for DM2. Cholelithiasis is a female dominated comorbidity (1.7, CI 1.5–2.0 and 1.5, CI 1.4–1.6). Cholecystitis is typically followed by DM2 in males. Pressure and non-pressure ulcers exhibit higher risks for DM1 (7.2, CI 5.2–9.9, and 7.4, CI 5.8–9.4) than DM2 patients (2.2, CI 2.0–2.4 and 4.2, CI 3.9–4.6). For males there are increased risks for disorders of prepuce (6.0, CI 3.5–10 and 3.1, CI 2.5–3.8), while for females there is increased risk for disorders of the urinary system (2.5, CI 2.2–2.8 and 1.8, CI 1.7–1.9). Evidence from epidemiological studies suggests that asymptomatic bacteriuria and symptomatic urinary tract infections occur more commonly in women with DM compared to non-diabetic controls [41, 46]. Increased prevalence of urinary incontinence and urge incontinence among women with DM2 [47, 48] has been reported.

**Limitations**. Only persons with inpatient stays were included in the study. To test if this pre-selection introduces a bias in our results, we repeated the study with a sample of all patients having been prescribed at least once a drug used in diabetes (ATC code starting with ‘A10’) in 2006 or 2007. We compare the frequencies of their diseases with those in the rest of the population, roughly 8.3 million patients. This assumes that DM patients with no hospital stay in the study period have no diagnosis and therefore no comorbidities. Although this is a highly incorrect assumption, it serves as a conservative test-assumption, which allows to test if the comorbidities are simply significant as a consequence of our limited sample that contains only inpatients. Results are shown in the supplement in S1 Fig. In the enlarged sample only one out of the 123 comorbidities using the inpatient sample has a *p*-value greater than 0.05 (M23), all other remain significant (*p*<0.05). Significance of comorbidity in the inpatient sample is therefore highly representative of comorbidity in the entire population. However, our approach might miss diabetic comorbidities that are typically not related to hospitalizations and that are most prevalent in younger patients, where the inpatient sample contains a lesser amount of the entire population, compare Fig. 1(a). Unknown pre-existing conditions may also affect the observed temporal order of the diseases, which has been addressed by applying a series of corrections to the lead/lag indicators, equations (2) and (3). Other limitations relate to the coding quality of disorders in the medical claims data, which has been shown to lead to an under-reporting of comorbidities [49] and may cause false negatives in our testing procedure.

This work shows the enormous potential that large-scale analyses of EHR data offer for the medical sciences. For the first time we develop a standardized testing procedure to obtain a complete comorbidity profile for DM1 and DM2 using medical claims data. This analysis is equivalent to 39 938 individual tests, each with the maximum number of patients available in a country. We identified 123 highly significant disorders with increased or decreased risks, strongly depending on patient age and sex. The comorbidities are investigated by a lead/lag analysis to inquire whether the relation between the diseases is more likely causal or consequential.

Taken together, these results underscore that there is a substantial number of disorders that are related to diabetes, besides the well-known long-term complications. Diabetic comorbidities are rule rather than exception and their treatment must address their high degree of age and sex dependence. Despite being a risk factor for certain diabetic complications, sex may also influence and to a certain degree even determine the mechanisms underlying the disease progressions. Our results may be of immediate use to improve screening practices and therapy of diabetic patients to increase their quality of life and potentially contribute to longer life expectancy due to early detection and treatment of important comorbidities. In particular we propose to screen and, where applicable, treat diabetes patients for comorbid depressions, since this allows a more efficient treatment of diabetes itself. Depressive patients should be screened for diabetes to detect it at an early stage and perform lifestyle interventions that focus on weight control. It is also important to treat depressive patients with drugs that have a minimum of side effects on weight gain, and lipid and glucose metabolism. Our results emphasize that physicians must be aware of non-traditional diabetic comorbidities and risk factors during anamnesis and that, for example, screening for diabetes may be appropriate in patients with cardiovascular diseases, CHF, or fatty liver, whereas diabetes patients should be screened for pancreatic cancer.

## Supporting Information

S1 Fig The values for relative risks (left panels) and sex ratios (right panels) for the 123 identified comorbidities are computed using a patient sample of all persons receiving a drug used in diabetes (ATC code A10). The results from the inpatient sample are reproduced to large parts, only disorder M23 exhibits non-significant *p*-values.
(TIF)

S1 Table ICD code and disease name for the 123 comorbidities identified in the co-occurrence analysis. For the age groups with the smallest *p*-value the relative risks *RR*, patient ages, and the corresponding *p*-values are shown for DM1 and DM2, respectively. Where the patient sample was too small to apply the statistical tests missing values are shown.
(PDF)

S1 Data Comorbidity data for DM1 patients, the relative risks *RR*_1_, the confidence intervals for *RR*_1_, if applicable the p-value for the co-occurrence analysis, and the sex ratio for each diagnosis and age group. (CSV)

S2 Data Comorbidity data for DM2 patients, the relative risks *RR*_2_, the confidence intervals for *RR*_2_, if applicable the p-value for the co-occurrence analysis, and the sex ratio for each diagnosis and age group. (CSV)
