# Supplementary figures and images for: Quantification of Diabetes Comorbidity Risks across Life Using Nation-Wide Big Claims Data
Source: PLoS Comput Biol. 2015 Apr 9;11(4):e1004125. doi: 10.1371/journal.pcbi.1004125 (PMC4391714; doi:10.1371/journal.pcbi.1004125)

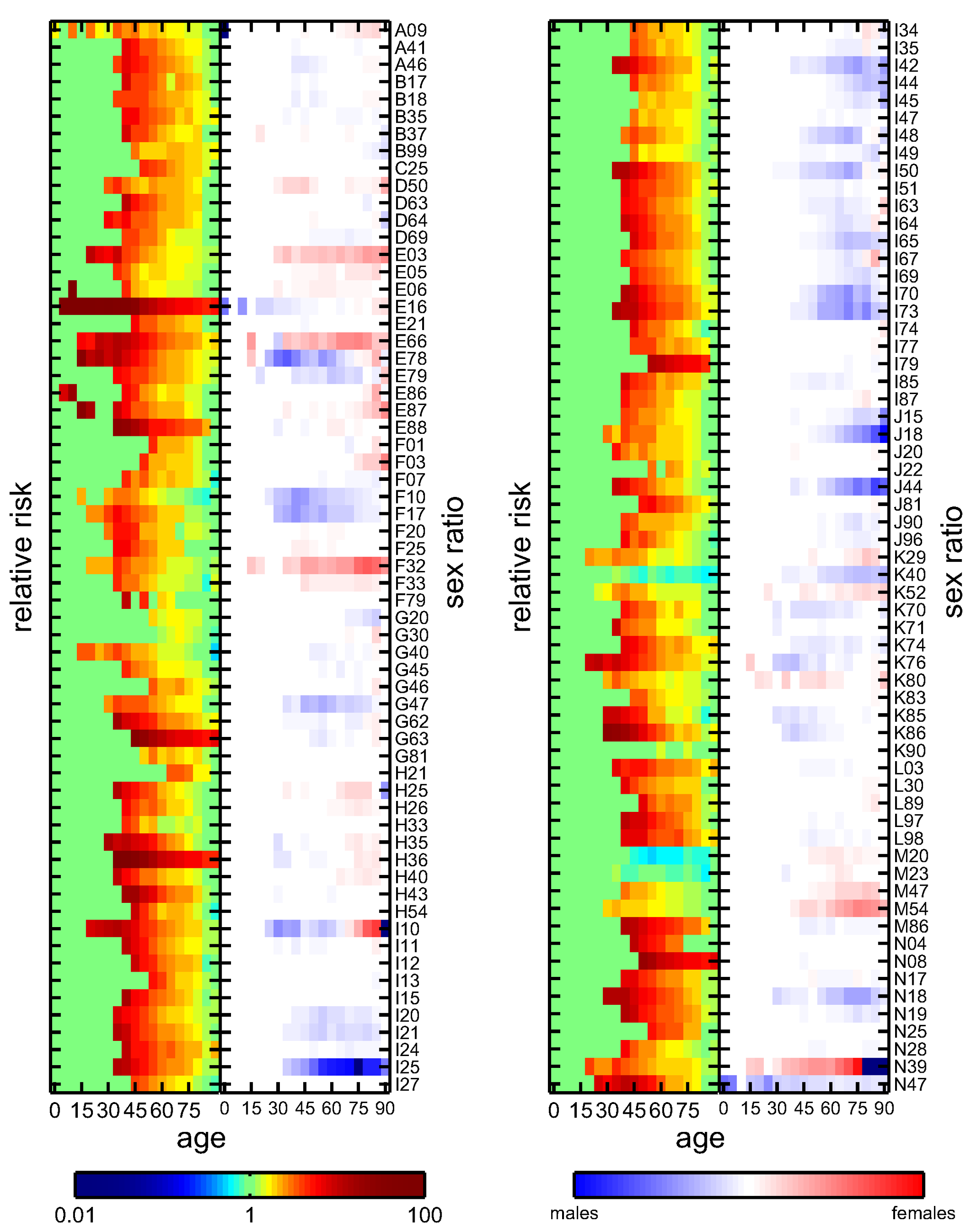

Supplement: S1 Fig — The results from the inpatient sample are reproduced to large parts, only disorder M23 exhibits non-significant p-values. (TIF) [file pcbi.1004125.s001.tif]
