# Supplementary material for: Quantification of Diabetes Comorbidity Risks across Life Using Nation-Wide Big Claims Data
Source: PLoS Comput Biol. 2015 Apr 9;11(4):e1004125. doi: 10.1371/journal.pcbi.1004125 (PMC4391714; doi:10.1371/journal.pcbi.1004125)
Supplement: S1 Table — For the age groups with the smallest p-value the relative risks RR, patient ages, and the corresponding p-values are shown for DM1 and DM2, respectively. Where the patient sample was too small to apply the statistical tests missing values are shown. (PDF) [file pcbi.1004125.s002.pdf]

Table S1: ICD code and disease name for the 123 comorbidities identified in the co-occurrence analysis. For the age groups with the smallest *p*-value the relative risks *RR*, patient ages, and the corresponding *p*-values are shown for DM1 and DM2, respectively. Where the patient sample was too small to apply the statistical tests missing values are shown.

		DM1			DM2		
**ICD**	**Disease**	***p***	***RR***	**age**	***p***	***RR***	**age**
A09	Diarrhoea and gastroenteritis of presumed infectious origin	0.004	4.4 (3.0-6.3)	45-50	0.0003	1.6 (1.5-1.7)	80-85
A41	Other septicaemia	$<10^{-4}$	12 (8.2-18)	45-50	$<10^{-4}$	2.7 (2.4-2.9)	65-70
A46	Erysipelas	0.0009	3.7 (2.9-4.8)	65-70	$<10^{-5}$	2.7 (2.5-3.0)	65-70
B17	Other acute viral hepatitis	0.006	7.1 (4.0-13)	50-55	0.002	6.9 (4.5-11)	40-45
B18	Chronic viral hepatitis	0.0007	6.3 (4.4-9.0)	50-55	0.0006	3.2 (2.6-3.8)	50-55
B35	Dermatophytosis	0.01	3.5 (2.4-5.2)	65-70	0.0005	2.6 (2.2-3.0)	65-70
B37	Candidiasis	0.002	5.5 (3.7-8.2)	55-60	0.002	2.1 (1.8-2.5)	65-70
B99	Other and unspecified infectious diseases	0.003	4.7 (3.3-6.9)	65-70	0.004	1.7 (1.5-2.0)	80-85
C25	Malignant neoplasm of pancreas	0.0006	8.6 (5.6-13)	55-60	0.0006	2.5 (2.1-2.8)	65-70
D50	Iron deficiency anaemia	0.0003	3.7 (3.0-4.6)	70-75	$<10^{-5}$	2.7 (2.4-2.8)	65-70
D63	Anaemia in chronic disease	$<10^{-4}$	6.3 (4.9-8.2)	65-70	$<10^{-4}$	2.8 (2.5-3.2)	65-70
D64	Other anaemia	$<10^{-5}$	4.9 (4.2-5.8)	65-70	$<10^{-6}$	2.6 (2.4-2.8)	65-70
D69	Purpura and other haemorrhagic conditions	0.009	3.6 (2.4-5.4)	55-60	0.001	2.0 (1.7-2.2)	65-70
E03	Other hypothyroidism	0.0001	9.3 (6.4-13)	35-40	0.0001	1.7 (1.6-1.9)	65-70
E05	Thyrotoxicosis	0.009	5.3 (3.1-8.9)	40-45	0.001	1.6 (1.5-1.8)	70-75
E06	Thyroiditis	$<10^{-6}$	38 (26-57)	10-15	0.04	1.7 (1.3-2.1)	55-60
E16	Other disorders of pancreatic internal secretion	$<10^{-15}$	170 (130-223)	20-25	$<10^{-8}$	5.4 (5.0-5.7)	80-85
E21	Hypoparathyroidism	0.0006	6.1 (4.4-8.6)	65-70	0.0002	3.0 (2.6-3.5)	67-70
E66	Obesity	$<10^{-6}$	3.7 (3.3-4.1)	65-70	$<10^{-16}$	4.8 (4.6-5.0)	55-60
E78	Disorders of lipoprotein metabolism and other lipidaemia	$<10^{-5}$	3.4 (3.0-3.7)	60-65	$<10^{-15}$	3.9 (3.7-4.0)	55-60
E79	Disorders or purine and pyrimidine metabolism	$<10^{-4}$	3.2 (2.8-3.7)	65-70	$<10^{-7}$	2.5 (2.4-2.6)	65-70
E86	Volume depletion	0.0001	9.4 (6.5-14)	10-15	0.0003	1.7 (1.6-1.8)	75-80
E87	Other disorders of fluid, electrolyte and acid-base balance.	$<10^{-4}$	5.9 (4.7-7.3)	60-65	$<10^{-5}$	2.5 (2.3-2.6)	65-70
E88	Other metabolic disorders	0.0001	6.4 (4.9-8.4)	60-65	$<10^{-7}$	5.1 (4.7-5.6)	60-65
F01	Vascular dementia	0.0008	3.0 (2.4-3.6)	75-80	$<10^{-4}$	2.1 (1.9-2.2)	75-80
F03	Unspecified dementia	0.001	2.5 (2.1-2.9)	75-80	$<10^{-4}$	1.8 (1.7-1.9)	75-80
F07	Personality and behavioral disorders due to known physiological condition	0.002	3.8 (2.9-5.1)	75-80	0.001	2.4 (2.0-2.7)	65-70
F10	Use of alcohol	0.02	2.3 (1.7-3.2)	40-45	0.0007	2.1 (1.9-2.4)	45-50
F17	Use of tobacco	0.0004	3.3 (2.7-4.1)	50-55	$<10^{-6}$	2.8 (2.6-3.0)	50-55
F20	Schizophrenia	0.09	2.1 (1.2-3.7)	65-70	0.001	3.4 (2.7-4.2)	45-50
F25	Schizoaffective disorders	-	-	-	0.003	4.3 (3.0-6.2)	40-45
F32	Depressive episode	0.001	2.3 (1.9-2.6)	65-70	$<10^{-4}$	1.7 (1.6-1.8)	65-70
F33	Recurrent depressive disorder	0.05	1.9 (1.3-2.6)	65-70	0.003	4.8 (3.3-7.0)	35-40
F79	Unspecified mental retardation	-	-	-	0.009	9.3 (6.0-15)	40-45

G20	Parkinson’s disease	0.002	2.3 (1.9-2.7)	75-80	0.001	1.5 (1.4-1.6)	75-80
G30	Alzheimer’s disease	0.04	1.7 (1.3-2.1)	75-80	0.001	1.5 (1.4-1.7)	75-80
G40	Epilepsy	0.004	4.6 (3.1-6.9)	35-40	0.003	1.6 (1.4-1.7)	65-70
G45	Transient cerebral ischaemic attacks	0.006	2.8 (2.1-3.7)	60-65	0.0005	1.7 (1.6-1.9)	65-70
G46	Vascular syndromes of brain in cerebrovascular diseases	0.002	3.9 (3.0-5.3)	80-85	0.012	1.9 (1.6-2.3)	70-75
G47	Sleep disorders	0.02	1.9 (1.5-2.4)	60-65	0.0001	2.3 (2.1-2.6)	50-55
G62	Other polyneuropathies	$<10^{-5}$	8.2 (6.6-10)	55-60	$<10^{-5}$	2.7 (2.5-3.0)	65-70
G63	Polyneuropathy in diseases c. e.	$<10^{-10}$	20 (17-23)	65-70	$<10^{-11}$	8.0 (7.6-8.4)	65-70
G81	Hemiplegia	0.008	3.9 (2.6-5.3)	65-70	0.001	1.9 (1.6-2.3)	65-70
H21	Other disorders of iris and ciliary body	0.001	10 (6.2-16)	75-80	0.006	6.2 (4.0-9.4)	55-60
H25	Senile cataract	$<10^{-4}$	4.7 (3.9-5.7)	55-60	0.0002	2.0 (1.8-2.2)	55-60
H26	Other cataract	0.001	5.2 (3.7-7.3)	55-60	0.004	2.3 (1.9-2.8)	55-60
H33	Retinal detachments and breaks	0.0003	18 (10-32)	30-35	0.05	2.3 (1.6-3.5)	45-50
H35	Other retinal disorders	$<10^{-6}$	10 (8-12)	55-60	$<10^{-6}$	4.3 (3.9-4.7)	55-60
H36	Retinal disorders in diseases c. e.	$<10^{-16}$	200 (160-250)	30-35	0.08	230 (180-310)	30-35
H40	Glaucoma	0.0002	14 (8.7-23)	40-45	0.003	1.9 (1.6-2.2)	60-65
H43	Disorders of vitreous body	$<10^{-6}$	36 (25-53)	45-50	$<10^{-4}$	4.9 (4.1-5.9)	55-60
H54	Blindness and low vision	0.01	4.6 (2.7-7.8)	65-70	0.002	3.0 (2.4-3.6)	65-70
I10	Essential (primary) hypertension	$<10^{-8}$	5.3 (4.8-5.9)	65-70	$<10^{-16}$	9.5 (8.8-10)	45-50
I11	Hypertensive heart disease	0.0005	3.6 (2.9-4.5)	60-65	$<10^{-7}$	2.9 (2.7-3.1)	65-70
I12	Hypertensive chronic kidney disease	0.0002	20 (12-35)	45-50	0.001	3.4 (2.8-4.1)	65-70
I13	Hypertensive heart disease and hypertensive renal disease	0.002	7.6 (4.7-12)	65-70	0.001	5.1 (3.9-6.6)	60-65
I15	Secondary hypertension	0.01	4.8 (2.7-8.3)	55-60	0.0006	11 (7.1-18)	40-45
I20	Angina pectoris	0.0007	3.2 (2.6-3.9)	55-60	$<10^{-7}$	2.3 (2.2-2.5)	65-70
I21	Acute myocardial infarction	$<10^{-4}$	3.2 (2.8-3.7)	75-80	$<10^{-7}$	2.9 (2.7-3.1)	65-70
I24	Other acute ischaemic heart diseases	$<10^{-4}$	6.6 (5.2-8.3)	65-70	$<10^{-4}$	3.1 (2.8-3.4)	65-70
I25	Chronic ischaemic heart disease	$<10^{-8}$	5.3 (4.7-5.9)	60-65	$<10^{-16}$	3.3 (3.2-3.4)	65-70
I27	Other pulmonary heart diseases	0.004	2.5 (2.0-3.1)	75-80	0.0002	2.4 (2.1-2.6)	65-70
I34	Nonrheumatic mitral valve disorders	0.001	2.4 (2.0-2.8)	75-80	$<10^{-5}$	2.3 (2.2-2.5)	65-70
I35	Nonrheumatic aortic valve disorders	0.004	2.7 (2.1-3.4)	65-70	$<10^{-4}$	1.8 (1.7-1.9)	75-80
I42	Cardiomyopathy	$<10^{-4}$	4.0 (3.3-4.7)	65-70	$<10^{-8}$	3.0 (2.8-3.2)	65-70
I44	Atrioventricular and left bundle-branch block	0.0004	5.1 (3.9-6.8)	60-65	$<10^{-5}$	2.5 (2.3-2.7)	65-70
I45	Other conduction disorders	0.007	3.1 (2.3-4.2)	70-75	0.0007	2.1 (1.9-2.4)	70-75
I47	Paroxysmal tachycardia	0.0004	3.3 (2.7-4.0)	75-80	0.0003	2.0 (1.8-2.2)	65-70
I48	Atrial fibrillation and flutter	$<10^{-4}$	2.1 (1.9-2.3)	75-80	$<10^{-9}$	2.0 (1.9-2.0)	75-80
I49	Other cardiac arrhythmias	0.008	2.2 (1.8-2.8)	70-75	0.001	1.5 (1.4-1.6)	75-80
I50	Heart failure	$<10^{-7}$	5.2 (4.7-5.9)	65-70	$<10^{-14}$	3.8 (3.6-3.9)	65-70
I51	Complications and ill-	0.0002	3.4 (2.8-4.0)	75-80	$<10^{-6}$	3.1 (2.8-3.4)	65-70

	defined descriptions of heart diseases.						
I63	Cerebral Infarction	0.0002	4.4 (3.5-5.6)	60-65	$<10^{-6}$	2.6 (2.4-2.8)	65-70
I64	Stroke, not specified as haemorrhage or infarction.	$<10^{-4}$	4.5 (3.7-5.5)	65-70	$<10^{-6}$	2.8 (2.6-3.1)	65-70
I65	Occlusion and stenosis of precerebral arteries.	$<10^{-4}$	5.3 (4.4-6.4)	60-65	$<10^{-8}$	3.0 (2.8-3.1)	65-70
I67	Other cerebrovascular diseases	0.0001	5.0 (3.9-6.2)	60-65	$<10^{-5}$	2.2 (2.1-2.3)	70-75
I69	Sequelae of cerebrovascular disease	0.005	3.5 (2.5-4.9)	60-65	$<10^{-6}$	3.0 (2.8-3.3)	65-70
I70	Atherosclerosis	$<10^{-8}$	6.0 (5.4-6.7)	70-75	$<10^{-11}$	3.0 (2.5-3.5)	65-70
I73	Other peripheral vascular diseases	$<10^{-7}$	5.7 (5.1-6.5)	65-70	$<10^{-13}$	3.9 (3.7-4.1)	65-70
I74	Arterial embolism and thrombosis	0.002	5.4 (3.7-7.7)	60-65	0.0004	3.0 (2.5-3.5)	60-65
I77	Other disorders of arteries and arterioles	0.001	10 (6.1-17)	50-55	0.0006	2.5 (2.2-2.9)	70-75
I79	D. of arteries, arterioles and capillaries in diseases c. e.	$<10^{-7}$	23 (18-29)	70-75	$<10^{-6}$	5.7 (5.2-6.1)	75-80
I85	Esophageal varices	0.005	4.3 (2.9-6.4)	55-60	0.0003	3.0 (2.5-3.5)	55-60
I87	Other disorders of veins	0.009	3.7 (2.5-5.5)	60-65	0.0004	2.3 (2.1-2.6)	65-70
J15	Bacterial pneumonia n. e. c.	0.02	2.5 (1.8-3.3)	70-75	0.0001	2.4 (2.2-2.7)	65-70
J18	Pneumonia, organism unspecified	$<10^{-4}$	2.7 (2.4-3.0)	75-80	$<10^{-6}$	2.3 (2.1-2.4)	65-70
J20	Acute bronchitis	0.03	1.9 (1.5-2.6)	80-85	0.0007	2.2 (2.0-2.5)	65-70
J22	Unspecified acute lower respiratory infection	0.02	3.4 (2.1-5.6)	75-80	0.002	3.1 (2.5-3.9)	65-70
J44	Other chronic pulmonary diseases	0.0003	2.9 (2.5-3.5)	55-60	$<10^{-8}$	2.2 (2.1-2.3)	65-70
J81	Pulmonary oedema	0.0003	9.9 (6.6-15)	60-65	$<10^{-4}$	3.7 (3.2-4.3)	65-70
J90	Pleural effusion, not elsewhere classified	0.0004	3.4 (2.1-5.6)	75-80	0.0001	3.1 (2.5-3.9)	65-70
J96	Respiratory failure, n. e. c.	0.001	9.4 (5.7-16)	10-15	$<10^{-4}$	2.3 (2.1-2.5)	65-70
K29	Gastritis and duodenitis	0.01	1.8 (1.5-2.1)	60-65	0.0002	1.5 (1.4-1.5)	70-75
K40	Inguinal hernia	0.04	0.37 (0.23-0.60)	75-80	0.001	0.48 (0.41-0.56)	65-70
K52	Other and unspecified noninfective gastroenteritis and colitis	0.006	2.1 (1.7-2.6)	75-80	0.0008	1.5 (1.4-1.6)	80-85
K70	Alcoholic liver disease	0.005	4.0 (2.7-5.7)	45-50	0.0001	2.6 (2.3-2.9)	55-60
K71	Toxic liver disease	0.05	2.8 (1.6-4.9)	55-60	0.0003	14 (8.5-23)	35-40
K74	Fibrosis and cirrhosis of liver	0.0004	5.0 (3.7-6.6)	55-60	$<10^{-4}$	2.4 (2.2-2.7)	65-70
K76	Other diseases of liver	0.0005	2.7 (2.2-3.1)	60-65	$<10^{-8}$	3.0 (2.8-3.2)	55-60
K80	Cholelithiasis	0.008	1,7 (1.5-2.0)	75-80	$<10^{-4}$	1.5 (1.4-1.6)	80-85
K83	Other diseases of biliary tract	0.003	7.5 (4.6-12)	50-55	0.03	1.4 (1.2-1.6)	80-85
K85	Acute pancreatitis	0.006	4.5 (3.0-6.9)	50-55	$<10^{-4}$	4.7 (3.9-5.8)	45-50
K86	Other diseases of pancreas	$<10^{-5}$	13 (9.3-17)	50-55	$<10^{-5}$	11 (8.4-14)	40-45
K90	Intestinal malabsorption	0.001	10 (6.3-17)	10-15	0.4	1.2 (0.7-2.2)	60-65
L03	Cellulitis and acute lymphangitis	0.0005	5.5 (4.0-7.4)	65-70	0.0006	2.5 (2.1-2.9)	65-70
L30	Other and unspecified dermatitis	0.01	5.1 (3.0-8.7)	45-50	0.001	1.9 (1.7-2.1)	80-85
L89	Decubitus ulcer	0.0002	7.2 (5.2-9.9)	65-70	$<10^{-4}$	2.2 (2.0-2.4)	80-85

L97	Ulcer of lower limb n. e. c.	$<10^{-4}$	7.4 (5.8-9.4)	65-70	$<10^{-6}$	4.2 (3.9-4.6)	65-70
L98	Other disorders of skin and subcutaneous tissue	$<10^{-5}$	9.0 (7.2-11)	70-75	$<10^{-4}$	3.4 (3.1-3.9)	65-70
M20	Acquired deformities of fingers and toes	0.09	0.44 (0.25-0.88)	60-65	0.002	0.41 (0.34-0.49)	65-70
M23	Internal derangement of knee	0.03	0.31 (0.19-0.52)	60-65	0.0006	0.45 (0.40-0.52)	65-70
M47	Spondylosis	0.04	1.7 (1.3-2.1)	85-90	0.0003	1.6 (1.5-1.7)	70-75
M54	Dorsalgia	0.06	1.3 (1.1-1.6)	65-70	0.002	1.4 (1.3-1.5)	60-65
M86	Osteomyelitis	$<10^{-6}$	13 (10-16)	65-70	$<10^{-5}$	4.4 (3.9-5.0)	65-70
N04	Nephrotic syndrome	$<10^{-4}$	33 (19-56)	45-50	0.001	4.9 (3.8-6.4)	60-65
N08	Glomerular d. in diseases c. e.	$<10^{-12}$	128 (98-166)	40-45	$<10^{-9}$	8.6 (8.2-9.1)	65-70
N17	Acute renal failure	$<10^{-4}$	13 (8.9-20)	45-50	$<10^{-6}$	3.4 (3.1-3.7)	65-70
N18	Chronic renal failure	$<10^{-10}$	8.0 (7.2-8.9)	65-70	$<10^{-14}$	4.2 (4.0-4.4)	65-70
N19	Unspecified renal failure	$<10^{-7}$	9.4 (8.1-11)	65-70	$<10^{-6}$	3.2 (2.9-3.5)	65-70
N25	Disorders resulting from impaired renal tubular function	0.002	9.0 (5.5-15)	70-75	0.001	3.9 (3.1-4.9)	65-70
N28	O. d. of kidney and ureter, n. e. c.	0.004	5.8 (3.7-9.1)	50-55	0.0008	2.7 (2.3-3.2)	55-60
N39	Other disorders of urinary system	$<10^{-4}$	2.5 (2.2-2.8)	80-85	$<10^{-7}$	1.8 (1.7-1.9)	80-85
N47	Disorders of prepuce	0.007	6.0 (3.5-10)	45-50	0.001	3.1 (2.5-3.8)	55-60
